# Treatment-free remission in chronic myeloid leukemia: the patient perspective and areas of unmet needs

**DOI:** 10.1038/s41375-020-0867-0

**Published:** 2020-05-26

**Authors:** Giora Sharf, Celia Marin, Jennifer A. Bradley, Zack Pemberton-Whiteley, Felice Bombaci, Rita I. O. Christensen, Bahija Gouimi, Nigel B. Deekes, Mina Daban, Jan Geissler

**Affiliations:** 1 CML Advocates Network, Bern, Switzerland; 2 Israeli CML Patients Organization, Netanya, Israel; 3 Quality Health Ltd, Derbyshire, UK; 4 Leukaemia Care, Worcester, UK; 5 Gruppo AIL Pazienti Leucemia Mieloide Cronica, Rome, Italy; 6 LyLe Patientforeningenen for Lymfekræft Leukæmi og MDS, Sealand Region, Denmark; 7 Association des malades atteints de leucémies AMAL, Marrakech, Morocco; 8 LMC France, Marseille, France; 9 LeukaNET e.V, Riemerling, Germany

**Keywords:** Chronic myeloid leukaemia, Quality of life

## Abstract

In CML, treatment-free remission (TFR) refers to having a stable deep molecular response without the need for ongoing tyrosine kinase inhibitor treatment. Whilst recommendations exist about the technical management of stopping and re-starting therapy, much is still unknown about the experiences of those considering and undertaking TFR. This study sought to obtain the patient perspective, identify areas of unmet needs and create recommendations for improvements. Fifty-six percent of patients reported fear or anxiety during treatment discontinuation, whereas only 7% of patients were asked if they needed psychological support during this period. Where patients re-initiated treatment; 59% felt scared or anxious, and 56% felt depressed. Twenty-six percent of re-initiated patients received psychological and/or emotional support at this time. Sixty percent of patients experienced withdrawal symptoms whilst discontinuing treatment, however, 40% of patients who experienced withdrawal symptoms reported that they were not fully supported by their doctor in managing all the symptoms. Healthcare professionals should further consider how they monitor the psychological well-being of patients who are discontinuing or re-initiating treatment, and review what support is offered in response to identified concerns. Surveillance of withdrawal symptoms should be a priority during treatment discontinuation, along with how healthcare professionals assist in the management of these.

## Introduction

Chronic myeloid leukemia (CML) is a myeloproliferative disorder that is most commonly characterized by the presence of a Philadelphia chromosome, caused by the genetic translocation t(9;22)(q34;q11). This genetic abnormality juxtaposes two genes (BCR and ABL1), whose fusion codes for the constitutively active tyrosine kinase is BCR-ABL1. Targeting this protein with tyrosine kinase inhibitors (TKIs) such as imatinib mesylate revolutionized treatment of this disorder [1]. The use of TKIs has fundamentally improved survival rates for the majority of patients with CML and many patients can expect a near normal life expectancy [2–5]. However, patients can experience TKI-related adverse side effects [6–9], toxicities [7, 10, 11], impact on quality of life (QoL) [6], and for some, the price of the drugs [12, 13]. Furthermore, TKIs are not recommended for female patients who are trying to conceive, during pregnancy or are breastfeeding [14].

In recent years, a number of studies have been conducted to demonstrate the possibility and safety of TKI cessation in well responding patients. The results of such studies have seen born the concept of treatment-free remission (TFR). Following a pilot study [15], two pioneer studies confirmed that treatment discontinuation was feasible and safe [16, 17], and numerous clinical trials and observational studies have been reported, not only after imatinib treatment [18–31], but also after second generation TKI (2GTKI) whether used in 1st or 2nd line [23–27, 32–37]. Recently, second attempts to discontinue TKI in patients who relapsed have been reported [32, 38, 39]. Recommendations on selection of patients with a higher probability of successful discontinuation have been proposed by global experts [40–43]. However, the point of view of the patients undergoing discontinuation of treatment is less frequently reported on.

The CML Advocates Network (CMLAN) is an active international network for leaders of Chronic Myeloid Leukemia (CML) patient groups. Its aim is to facilitate and support best practice sharing among patient advocates across the world. CMLAN’s objectives in this research were to inform the development of a range of guides for patients and healthcare professionals, that consider the patient experience of discontinuing treatment. The research did not seek to replicate the formal collection of scientific data such as prognosis, or clinical requirements for stopping treatment, as this has been expertly collected through existing medical studies and clinical trials. The CMLAN study was explicitly designed to focus on the patient perspective. Therefore, the areas of investigation include subjects less frequently covered in existing scientific research, but have importance to patients. These include, but are not limited to: the patient’s reasons for discontinuing treatment, the emotional impact within the TFR journey, and what information and support was provided. Through analysis of the survey data, it was identified that there were areas within the TFR journey where opportunities exist to improve patient care and experience. This manuscript summarizes and evidences these findings.

## Methods

### Questionnaire design

The questionnaire was designed by an expert panel of eight CML patients and went through two rounds of testing. Information was collected across five sections: about you (demographics); considerations around stopping treatment; experiences during the first six months of stopping treatment; restarting treatment; and, ongoing long-term TFR. Patients only completed the sections that were relevant to their experiences of stopping treatment. The demographic section collected data on relevant CML patient characteristics, including gender, age and country of residence. Consideration of stopping treatment was classified as “Phase I” of the stopping treatment journey. The questions collected information about the considerations respondents took before attempting, or rejecting TFR. Patients’ experiences during the first 6 months of discontinuing treatment were classified as “Phase II”. Most molecular recurrences (relapses) happen within the first 6 months after stopping treatment, therefore this stage of the stopping process is also known as the probation phase. This section collected information on respondents’ experiences during the probation phase. The restarting treatment section was classified as “Phase IIIA” and collected information from the respondents who had stopped treatment, but subsequently had to restart. The experiences of those in long-term remission beyond the 6-month probation phase were classified as “Phase IIIB”. Questions here included information on the concerns respondents still have and what they would change about their experience of discontinuing treatment.

Across all sections of the questionnaire, the aim was to ask questions that allowed insight and understanding into what the patient had experienced, rather than the clinical perspective. Due to this focus on patient perspective, it was the authors’ opinion that the views of patients discontinuing treatment within the context of a clinical trial, were no more or less important than patients who discontinue in “real life”. Consequently, information around if respondents had participated in TFR clinical trials was not collected as part of this study. It is not believed that the conclusions are biased or weaker due to the lack of this data.

### Administration

Administration of the survey was through a web-based questionnaire, between 14th March 2018 and 1st August 2018. The questionnaire was made available in 11 languages: Arabic, Danish, English, Finnish, French, German, Hebrew, Italian, Japanese, Russian and Spanish. The survey was promoted by the CMLAN, across 12 CML patient organization websites; CMLAN social media channels (Facebook, Twitter, Instagram, LinkedIn,); 64 CMLAN members’ Facebook pages; six emails to CMLAN members; three newsletters to CMLAN members; at the European Hematology Association Congress 2018; and, at the CML Horizons 2018 Conference. The project elicited 1016 responses from patients across 68 countries.

Anecdotal evidence from other survey programmes suggests that patients who are already engaged in wider discussions about treatment options (for example, through patient advocacy organizations), are more likely to complete questionnaires; and that because of this engagement (for which we can find no scientific evidence) they may report either better (because they are better informed) or worse (because poor treatment led them to seek help) experiences. In the survey design for this study, there is an explicit assumption that the experience of patients stopping treatment is not statistically associated with their involvement with patient advocacy organizations, or their desire to engage in completing a questionnaire—and therefore that there is no inherent bias in the methodology or results.

### Analysis of responses

For all questions (except for those asked in the form of “tick all that apply”) the percentage responses are calculated after excluding those respondents that did not answer that question. All percentages are rounded to the nearest whole number. When added together, the answers to a particular question may not total 100% because of this rounding. Figures have been calculated excluding responses where the question was not applicable to the respondent’s circumstances, or they felt unable to give a definite answer. The base size for questions that have been asked as “tick all that apply” is determined by the number of eligible respondents. As such, the missing count for a “tick all that apply” response option represents any eligible respondents who have chosen not to select that particular option.

### Limitations

The survey has several limitations. The survey was only available online. Whilst there were benefits to this methodology, it will have introduced limitations to accessibility by factors such as region and socioeconomic status. Respondents were self-selected, participated on a voluntary basis and were recruited through patient organizations, therefore cannot reflect the perspectives of all CML patients.

## Results

### Demographics

1016 responses were collected across 68 countries. Fifty-five percent (*n* = 550) of respondents were female, the median age was 53 (with a range of 16–92); education levels were varied. Respondents were grouped into the designated World Health Organization (WHO) regions; 56% (*n* = 563) were from countries assigned to the European region. Sixty percent (*n* = 608) of respondents reported that their main place of treatment was a hospital, and 39% (*n* = 389) of respondents had been living with CML for 10 years or more. Forty-nine percent (*n* = 494) of respondents proceeded to stop treatment. Full demographics of respondents at each phase are shown in Table 1. Fifty-one percent (*n* = 242) of respondents who stopped treatment reported that the medication they were taking before stopping was imatinib. Full range of responses are shown in Table 2.

**Table 1:** Demographics of the respondents.

Variables	Phase I	Phase II	Phase IIIA	Phase IIIB
	*N* %	*N* %	*N* %	*N* %
Gender				
Male	453 (45)	196 (40)	51 (32)	98 (49)
Female	550 (55)	293 (60)	106 (68)	103 (51)
Age (at the time when the survey was completed)				
Median, range (years)	53 (16–92)	56 (21–92)	56 (26–82)	57 (21–85)
Education				
No formal qualifications	50 (5)	19 (4)	10 (6)	2 (1)
High school qualifications or High school diploma	228 (23)	107 (22)	30 (19)	48 (24)
University—Bachelors or Undergraduate degree	361 (36)	160 (33)	44 (28)	62 (31)
University—Masters or PHD	208 (21)	112 (23)	41 (26)	45 (22)
Career or technical qualifications	160 (16)	92 (19)	32 (20)	44 (22)
WHO Region (grouped from country of residence when the survey was completed)				
Africa (AFRO)	13 (1)	2 (0)	0 (0)	1 (0)
Eastern Mediterranean (EMRO)	27 (3)	9 (2)	3 (2)	3 (1)
Europe (EURO)	563 (56)	330 (67)	111 (70)	132 (66)
Pan Americas (PAHO)	171 (17)	79 (16)	23 (15)	36 (18)
South-East Asia (SEARO)	33 (3)	3 (1)	0 (0)	2 (1)
Western Pacific (WPRO)	198 (20)	67 (14)	21 (13)	27 (13)
Place of treatment (tick all that apply)				
Community/family doctor	52 (5)	26 (5)	6 (4)	15 (7)
Hospital	608 (60)	255 (52)	85 (53)	87 (43)
Specialist all cancers center	221 (22)	120 (24)	43 (27)	49 (24)
CML centre of excellence	214 (21)	135 (27)	43 (27)	65 (32)
Other	53 (5)	26 (5)	8 (5)	12 (6)
Time after diagnosis of CML				
Less than 1 year	34 (3)	3 (1)	1 (1)	1 (1)
1–3 years	120 (12)	8 (3)	2 (1)	2 (1)
3–5 years	140 (14)	49 (10)	14 (9)	13 (7)
5–10 years	308 (31)	183 (38)	66 (43)	62 (32)
10 years or more	389 (39)	237 (49)	72 (46)	115 (60)
Discontinued treatment				
Yes	494 (49)			
No	522 (51)

**Table 2:** CML treatment.

Variables	Not discontinued	Discontinued
	*N* %	*N* %
Medication		
Imatinib (Glivec/Gleevec or generic Imatinib)	263 (56)	242 (51)
Nilotinib (Tasigna)	100 (21)	141 (29)
Dasatinib (Sprycel)	88 (19)	75 (16)
Bosutinib (Bosulif)	9 (2)	5 (1)
Ponatinib (Iclusig)	3 (1)	1 (0)
Interferon Alpha	0 (0)	14 (3)
ABL001/Asciminib	0 (0)	0 (0)
Other	9 (2)	1 (0)

### Key findings in Phase I—the consideration phase

All 1016 respondents were asked to complete the Phase I section of the questionnaire.

Most respondents reported first hearing about the possibility of stopping treatment through a healthcare professional 49% (*n* = 476), followed by 21% (*n* = 209) who heard through a patient organization. When asked to select the main reasons that made them consider attempting TFR, 51% (*n* = 516) said they wanted to get rid of current treatment side effects, and 48% (*n* = 488) wanted to see if they could be free of CML without therapy. Seventeen percent (*n* = 168) reported they considered stopping because their doctor proposed they join a “stopping treatment” clinical trial. The full range of responses is shown in Table 3.

**Table 3:** Motivation for discontinuing treatment.

Variables	*N* %
Where first heard about the possibility of stopping treatment	
Healthcare professional	476 (49)
Patient organization	209 (21)
Family	4 (0)
Printed materials (e.g. brochures or leaflet)	7 (1)
Media (e.g. scientific articles or lay press)	28 (3)
Internet	120 (12)
Social media (e.g. Facebook or web based group)	81 (8)
Other	51 (5)
Main reasons for considering stopping treatment (tick all that apply)	
To get rid of current treatment side effects	516 (51)
The fear of side effects caused by long-term treatment	429 (42)
Not needing to take medication everyday	378 (37)
To see if can be free of CML without therapy	488 (48)
Doctor proposed joining a “stopping treatment” clinical trial	168 (17)
Financial reasons—reduction of costs	98 (10)
Planned or unplanned pregnancy	105 (10)
Other	72 (7)

Respondents were asked which topics they discussed with their doctor whilst making their decision to try stopping treatment: 60% (*n* = 606) of respondents discussed risks of stopping; 50% (*n* = 513) discussed the requirements to be met in order to stop; 48% (*n* = 490) discussed the benefits of stopping; 34% (*n* = 346) discussed the timing of when to stop; 21% (*n* = 215) discussed the drug withdrawal symptoms. Fourteen percent (*n* = 143) did not have a discussion with their doctor. Following the discussion with their doctor, 55% (*n* = 555) of respondents said they still had concerns about TFR being unsuccessful, and their disease reoccurring.

Respondents were asked to indicate the reasons that made them worry about stopping treatment. Fifty-seven percent (*n* = 579) reported recurrence of CML; 20% (*n *= 203) said they would not feel safe going off treatment; 16% (*n* = 158) did not have enough information about stopping treatment; 12% (*n* = 120) had a fear of withdrawal symptoms; 7% (*n* = 70) said there is a lack of proper quality PCR monitoring. Twenty-six percent (*n* = 261) reported that they were not worried about stopping treatment.

Seventy-three percent (*n* = 591) of respondents said their doctor supported their decision to try stopping treatment. Sixty-one percent (*n* = 624) of respondents reported they received support and information about stopping treatment from their doctor or another healthcare professional; 32% (*n* = 327) said they received it from a patient organization; 30% (*n* = 303) from other CML patients who have stopped treatment; and, 29% (*n* = 299) from the internet.

When asked what information about stopping treatment and TFR they would have liked to have received: 52% (*n* = 533) of respondents would have liked information on results from clinical trials; 49% (*n* = 499) general information on all the steps of the stopping treatment journey; 44% (*n* = 452) the expectations of the risks and opportunities of stopping treatment; 40% (*n* = 407) the withdrawal effects after stopping; 36% (*n* = 368) the side effects on restarting therapy; 32% (*n* = 330) the required PCR monitoring; and, 22% (*n* = 227) the psychological effects.

### Key findings in Phase II—the probation phase

At the time of the study, no (published) consensus concerning the minimal requirements for TFR had been reached. Subsequent recommendations published by European LeukemiaNet [43] advise minimal requirements for TFR that include duration of TKI therapy >5 years (>4 years for 2GTKI) and duration of deep molecular response (DMR) (MR^4^ or better) >2 years. Seventy-seven percent (*n* = 359) of respondents reported that they had been on CML medication for ≥5 years, the median was 7 years (with a range of 1–27). Seventy-eight percent (*n* = 382) said they had been in DMR of at least MR^4^, or BCR-ABL transcript levels below 0.01%, for over 2 years before stopping. Table 4 illustrates the full range of responses.

**Table 4:** Duration of treatment and deep molecular response reported by respondents to Phase II.

Variables	*N* %
**Time on CML treatment (years)**	
1	8 (2)
2	15 (3)
3	44 (9)
4	40 (9)
5	61 (13)
6	33 (7)
7	42 (9)
8	47 (10)
9	35 (8)
10	34 (7)
11	19 (4)
12	25 (5)
13	22 (5)
14	21 (5)
15	9 (2)
16	2 (0.4)
17	2 (0.4)
18	1 (0.2)
19	1 (0.2)
24	2 (0.4)
25	1 (0.2)
26	1 (0.2)
27	1 (0.2)
**Time in deep molecular response (DMR) in years**	
*DMR defined as at least MR*^*4.0*^	
Not in DMR	7 (2)
< 1	16 (3)
1–2	55 (12)
2–3	90 (20)
3–4	84 (18)
4–8	124 (27)
>8	84 (18)

Fifty-six percent (*n* = 266) of respondents said that they “completely” had all the information they wanted when they stopped treatment. When asked what topics they discussed with their doctor during Phase II, 39% (*n* = 168) of respondents reported having a discussion on how to deal with withdrawal symptoms. Forty-seven percent (*n* = 222) reported that their doctor or another healthcare professional asked them if they were experiencing any physical withdrawal symptoms whilst stopping treatment; 27% (*n* = 128) were not asked, but would have liked to have been; and 26% (*n* = 121) were not asked but did not feel it was necessary. Sixty percent (*n* = 288) of respondents reported experiencing withdrawal symptoms; of these, 32% (*n* = 91) said they continued for a few months after stopping. Of those experiencing withdrawal symptoms: 89% (*n* = 257) reported withdrawal pain in muscles, joints or bones; 51% (*n* = 147) tiredness; 26% (*n* = 76) depressive episodes, fear or bad mood; 26% (*n* = 74) sweating or skin problems; and, 11% (*n* = 31) weight loss. Forty percent (*n* = 112) of the respondents who experienced side effects, reported that their doctor did not support them in managing all their physical withdrawal effects during discontinuation of treatment.

**Fig. 1:**
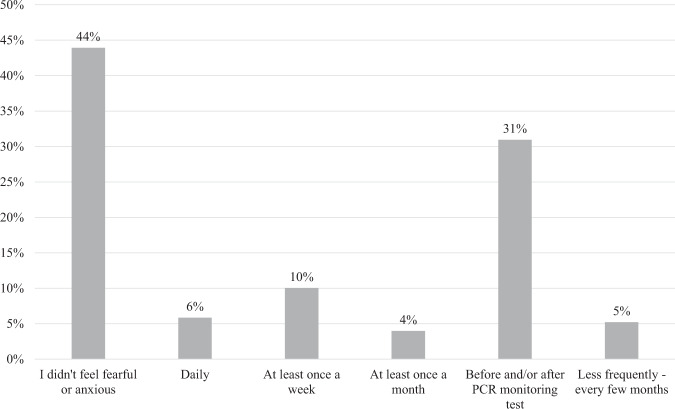
Frequency of fear or anxiety during Phase II. This chart illustrates how patients responded when asked how frequently they experienced fear or anxiety during Phase II.

When asked what topics they discussed with their doctor during the stopping phase, 18% (*n* = 69) of respondents reported having a discussion on how to deal with psychological aspects. Seventy-four percent (*n* = 268) reported that one of the benefits of stopping treatment was that it had a positive impact on their emotional wellbeing, however, 56% (*n* = 268) of respondents said that they felt fear or anxiety at some point during the stopping phase, and 55% (*n* = 148) of these reported this happened around the time of their PCR monitoring tests. Figure 1 illustrates the full range of responses.

Seven percent (*n* = 32) of respondents reported that their doctor asked them if they needed psychological support whilst stopping treatment; 26% (*n* = 120) were not asked but would have liked to have been; 67% (*n* = 312) were not asked, but did not feel it was necessary. Twenty percent (*n* = 93) of respondents reported that they did receive psychological and/or emotional support during discontinuation of treatment; 23% (*n* = 110) did not receive any, but would have liked to; and 57% (*n* = 269) did not receive any, but felt it was not necessary. Of the respondents who said they had received psychological and/or emotional support: 71% (*n* = 66) reported receiving support from friends and family; 26% (*n* = 24) from patient organization/s; 25% (*n* = 23) from a social media group; and, 20% (*n* = 19) received counseling.

During Phase II, 92% (*n* = 426) of respondents reported having a discussion on how often to monitor their BCR-ABL transcript levels, 76% (*n* = 339) discussed response levels and when/if to restart treatment and 70% (*n* = 294) discussed the time taken to receive the results of the last PCR test. Sixty-four percent (*n* = 308) of respondents reported that on average they were monitored monthly by their doctor through PCR tests during the probation phase, and 34% (*n* = 165) were monitored less frequently.

### Key findings in Phase IIIA—the restarting treatment phase

Of the 494 respondents who reported having stopped treatment, 32% (*n* = 159) reported that their disease had reoccurred, and treatment had to restart. Seventy-seven percent (*n* = 119) of patients reported that their disease reoccurred within the first 6 months of stopping treatment.

When they were first told their disease had reoccurred: 59% (*n* = 81) of respondents strongly agreed/agreed that they felt scared/anxious; 91% (*n* = 138) strongly agreed/agreed that they felt disappointed; 56% (*n* = 74) strongly agreed/agreed that they felt depressed; 20% (*n* = 25) strongly agreed/agreed that they felt confused; and, 4% (*n* = 5) strongly agreed/agreed that they felt relieved.

When asked how they felt emotionally in the first few weeks after restarting treatment, 35% (*n* = 55) of respondents reported feeling emotionally worse at this point compared with before stopping, and 30% (*n* = 48) reported feeling emotionally worse than during stopping treatment.

Respondents were asked to rate side effects by the extent they affected their everyday life in the weeks before stopping treatment, and then again when they restarted treatment. A score of 1 indicated that the side effect mildly affected their everyday life, and 5 indicated that the side effect completely affected their everyday life. No response indicated they did not experience the side effect. A mean score was calculated for each side effect. The side effects that showed the biggest mean score increase in impact between before stopping and restarting were feeling sad, feeling distressed, and anxiety (Table 5).

**Table 5:** Severity of side effects reported by Phase IIIA respondents, before stopping and after restarting treatment.

Variables	N experienced before stopping treatment	% experienced before stopping treatment	$\overline {\mathrm{X}}$ severity score before stopping treatment	N experienced after restarting treatment	% experienced after restarting treatment	$\overline {\mathrm{X}}$ severity score after restarting treatment	Difference of $\overline {\mathrm{X}}$ severity score after restarting treatment	% difference of $\overline {\mathrm{X}}$ severity score after restarting treatment
**Side effect**								
Feeling sad	85	(53)	2.14	86	(54)	2.73	0.59	(28)
Feeling distressed (upset)	83	(52)	2.34	84	(53)	2.90	0.57	(24)
Anxiety	84	(53)	2.31	91	(57)	2.80	0.49	(21)
Headaches	81	(51)	2.14	77	(48)	2.48	0.34	(16)
Lack of appetite	70	(44)	1.53	66	(42)	1.77	0.24	(16)
Disturbed sleep	98	(62)	2.80	96	(60)	3.03	0.24	(08)
Diarrhoea	89	(56)	2.12	81	(51)	2.31	0.19	(09)
Feeling of malaise (not feeling well)	88	(55)	2.63	86	(54)	2.79	0.17	(06)
Hair loss	89	(56)	2.31	76	(48)	2.46	0.15	(06)
Bruising	77	(48)	1.57	74	(47)	1.69	0.12	(07)
Difficulty thinking clearly	89	(56)	2.44	92	(58)	2.53	0.09	(04)
Vomiting	73	(46)	1.48	63	(40)	1.57	0.09	(06)
Nausea	88	(55)	2.38	82	(52)	2.46	0.09	(04)
Menstrual cycle issues	69	(43)	1.83	55	(35)	1.85	0.03	(02)
Shortness of breath	95	(60)	2.44	80	(50)	2.46	0.02	(01)
Pain	89	(56)	2.72	79	(50)	2.72	0.00	(0)
Fatigue (tiredness)	125	(79)	3.30	121	(76)	3.31	0.00	(0)
Dry mouth	81	(51)	2.22	78	(49)	2.22	0.00	(0)
Rash or skin change	95	(60)	2.69	82	(52)	2.67	−0.02	−(01)
Numbness or tingling	90	(57)	2.34	74	(47)	2.31	−0.03	−(01)
Remembering things	94	(59)	2.54	83	(52)	2.48	−0.06	−(02)
Eye bleeds	90	(57)	1.86	77	(48)	1.77	−0.09	−(05)
Swelling of hands, feet, abdomen, and around eyes	101	(64)	2.86	91	(57)	2.65	−0.21	−(07)
Skin pigment changes	84	(53)	2.52	70	(44)	2.29	−0.24	−(09)
Muscle soreness or cramping	113	(71)	3.25	103	(65)	2.97	−0.28	−(09)
Other	16	(10)	2.63	14	(09)	1.50	−1.13	−(43)

Twenty-six percent (*n* = 40) of respondents reported that they did receive psychological and/or emotional support during the restarting of treatment; 25% (*n* = 39) did not receive any, but would have liked to; and, 48% (*n* = 74) did not receive any, but felt it was not necessary. Of the respondents who said they had received psychological and/or emotional support: 85% (*n* = 34) reported receiving support from friends and family; 25% (*n* = 10) received counseling; 20% (*n* = 8) reported receiving support from a social media group; and, 18% (*n* = 7) received support from patient organization/s.

### Key findings in Phase IIIB—the long-term remission phase

Sixty-seven percent (*n* = 132) of respondents reported feeling that overall, they receive adequate care in the long-term TFR phase; 30% (*n* = 60) reported that this happens to some extent; and, 3% (*n* = 6) said that they do not receive adequate care.

When asked how they felt their doctor could improve their experience of stopping treatment, 47% (*n* = 95) of respondents reported they felt no improvement was necessary; 32% (*n* = 65) wanted more information on current data in easy to understand language; 11% (*n* = 23) wanted better PCR monitoring; 10% (*n* = 21) wanted a better doctor/patient relationship; and, 10% (*n* = 21) wanted better psychological support.

Respondents reported the major concerns they had while in Phase IIIB. Fifty-eight percent (*n* = 118) reported that late reoccurrence of CML was a major concern; 34% (*n* = 69) had uncertainty about the future in terms of CML; 29% (*n* = 58) were concerned over the misunderstanding of people thinking that they are cured; 26% (*n* = 52) had concerns over late detection of a reoccurrence; and, 4% (*n* = 9) were concerned that they have more frequent PCR tests than before stopping. Twenty-eight percent (*n* = 57) reported not having any concerns.

Twenty-five percent (*n* = 49) of respondents reported that they do receive psychological and/or emotional support during the long-term remission phase; 16% (*n* = 32) do not receive any, but would like to; and, 59% (*n* = 116) do not receive any, but feel it is not necessary.

Of the respondents who said they receive psychological and/or emotional support: 76% (*n* = 37) reported receiving support from friends and family; 33% (*n* = 16) from a social media group; 31% (*n* = 15) from patient organization/s; and, 16% (*n* = 8) receive counseling.

### Advice from patients

All patients (*n* = 494) who attempted stopping treatment (whether successful or not) were asked what their advice would be to other CML patients who are considering stopping treatment. Seventy-nine percent (*n* = 392) advised to always be well informed about your PCR results and treatment options; 69% (*n* = 342) advised to look for the best doctor with experience in stopping treatment; 59% (*n* = 290) advised to talk with other patients who have stopped, or are considering stopping; 51% (*n* = 254) advised to receive information from patient organizations about stopping treatment; 50% (*n* = 248) advised to look for simple and good information about each step of stopping treatment; 33% (*n* = 162) advised to get emotional support; and, 25% (*n* = 125) advised to get psychological support.

## Discussion and conclusion

To our knowledge this is the largest study conducted to gain knowledge about undertaking TFR that is taken purely from the perspective of the patient. There is existing research on patient perceptions of TFR [44–47], however, these focus on motivation and concerns about undertaking TFR. We believe this study is one of a limited number that investigates the experience of patients throughout all stages, and in particular looks at the support and management of the process beyond that of the management of PCR monitoring.

### Psychological and emotional impact of TFR

Our results evidence that respondents in the probation phase particularly felt fear or anxiety around the time of their PCR monitoring tests, an occurrence that is often dubbed “PCR-itis” (or scanxiety, in solid tumors) and is known to affect patients with cancer [48]. The implications of test results for TFR patients are perhaps magnified when they are not on treatment, as not only is there the potential to “fail” at TFR, but to “lose” a level of major molecular response that had previously been maintained for many years. With recommendations stating that monitoring should take place monthly in this phase [41–43], there is the potential for fear and anxiety to be frequent at this point in the TFR journey. However, only a small proportion of respondents said they discussed how to deal with psychological aspects with their doctor, and even fewer said that their doctor asked them if they needed psychological support during this phase.

The data also suggests that the requirement to restart treatment can have a strong negative psychological impact on patients, and that the desire for psychological support will be greater in the restarting treatment phase. In Phase IIIA, when they were first told they had molecular reoccurrence, many respondents reported feeling scared/anxious, and depressed. In addition, there were respondents who reported feeling emotionally worse during this phase than before discontinuation, and during discontinuation of treatment. There was also an increase in the reported severity of the impact of the emotional side effects of anxiety, feeling distressed and feeling upset in this phase, compared with before discontinuation of treatment. These results echo those of Sogawa et al. [49], who reported significantly higher anxiety and depression on the Hospital Anxiety and Depression Scale (HADS), at reintroduction of TKI than at the point of TKI discontinuation.

Despite the reported negative impact on psychological and emotional wellbeing, the data shows there were patients who wanted emotional support during the probation and restarting phases but did not receive it.

Whilst psychological support seems to be less of an issue in Phase IIIB (long-term TFR, ongoing past the probation period), late reoccurrence was reported as a major concern by a majority of respondents.

The survey did not collect information on what specifically caused concerns during the probation and restarting phases, and further investigation into this would be beneficial for the healthcare and wider CML communities.

This study’s findings support existing recommendations [50] that psychological wellbeing of CML patients attempting TFR should be a consideration of healthcare professionals and form part of routine monitoring.

### TKI withdrawal

Many of the respondents who stopped treatment reported that they experienced withdrawal symptoms. However, results indicate that patients did not always discuss the possibility of drug withdrawal symptoms with their doctor before making their decision to stop treatment. During the probation phase there were patients who did not have a discussion with their doctor about how to deal with withdrawal symptoms. Furthermore, during the discontinuation of treatment not all patients were asked if they were experiencing withdrawal effects, and many of these would have liked to have been. Where respondents experienced physical withdrawal symptoms during the discontinuation of treatment, results indicate there are instances where there could be further doctor support in the management of these. With clinical study data suggesting that up to 30% of patients who stop treatment will experience withdrawal symptoms [35, 51], there is a strong indication that importance needs to be placed on the issues of support and management of TKI withdrawal.

## Conclusion

Our research indicates that there are points in the TFR journey where patients do not always get all the advice, information or support they want around psychological issues and TKI withdrawal.

Results show that patients in the probation and restarting phases are likely to experience a negative impact on their mental well-being in the form of fear, worry, depression or anxiety. However, doctors do not always discuss the potential impact discontinuing treatment may have on a patient’s mental well-being, or ask them if they need psychological support. It is our recommendation that doctors should discuss mental health with patients early in the consideration and/or probation phases, so they are aware of and prepared for changes. Doctors should decide with the patient if it is appropriate to monitor their mental health, particularly during the probation period and treatment re-initiation. Further investigation into how mental health can be monitored during the TFR journey should be a consideration of future studies.

In addition, it is our recommendation that doctors should discuss what psychological support may be suitable and/or available for patients. While formal medical psychological intervention may not be necessary, signposting to other sources of support should be considered.

Research shows that TKI withdrawal is a possibility for patients who discontinue treatment. However, our results show that this is not always discussed when patients are making their decision to attempt TFR. In addition, once patients have discontinued treatment they are not always asked if they are experiencing withdrawal symptoms or offered support to manage them. It is our recommendation that healthcare professionals should address this by informing patients of the potential for withdrawal symptoms early in the TFR decision process. Once treatment has been discontinued, doctors should monitor for known withdrawal symptoms, encourage patients to report any symptoms they experience, and offer advice and support to on how to manage these.

### Supplementary information

TFR Patient Experience Questionnaire - English

## Data Availability

The data that support the findings of this study are available from the corresponding author on request.
