# Supplementary material for: Treatment-free remission in chronic myeloid leukemia: the patient perspective and areas of unmet needs
Source: Leukemia. 2020 May 26;34(8):2102–12. doi: 10.1038/s41375-020-0867-0 (PMC7387306; doi:10.1038/s41375-020-0867-0)
Supplement: Supplementary file 1 — TFR Patient Experience Questionnaire - English [file 41375_2020_867_MOESM1_ESM.pdf]

Introduction

This questionnaire is about your experiences of before and after stopping treatment, and attempting to achieve a Treatment Free Remission with CML.

The anonymised results of the survey will be used by the CML Advocates Network, the global CML umbrella organisation for all CML patient organisations, to provide a set of information packs and documents to its member organisations. These will include information for patients on all stages of considering and stopping treatment for CML, and for doctors, to inform them about patients' concerns, considerations and needs throughout all stages of considering and stopping treatment for CML.

Taking part in this survey is voluntary.
Published reports will not contain any personal details.

IMPORTANT INFORMATION

To make sure the information we collate is useful, we need to collect some personal details from you. The purpose of collecting this information is to generate aggregated statistics about the care and treatment people receive.

By completing this questionnaire, you are giving your consent for the information you provide to be used for the above purposes.

No names, emails address, or identifying information will be included in any publications or presentations based on these data, and your responses to this survey will remain confidential.

If you have any queries about the questionnaire, please email TFR4CML@quality-health.co.uk

About the questionnaire

Who should complete the questionnaire?
The questions should be answered by you, as the person who has been diagnosed with CML. If you need help to complete the questionnaire, the answers should be given from your point of view – not the point of view of the person who is helping.

The questionnaire is split into five sections:

- The first part of the questionnaire is '**About you'**. In this section, we want to know some background information about you and your CML treatment
- The second part of the questionnaire is called '**Phase I – Considerations around stopping treatment'**. In this section, we would like to find out what considerations and discussions took place when you were deciding if you should stop treatment
- The third part of the questionnaire is called '**Phase II – Probation period'**. This section is about your experiences during the first 6 months (probation period) of stopping treatment
- The fourth part of the questionnaire is called '**Phase IIIA – Restarting treatment**'. If your disease reoccurred we would like to know about your experiences of restarting treatment again
- The fifth part of the questionnaire is called '**Phase IIIB – Long term remission**'. If you are still in Treatment Free Remission we would like to know a little bit about your experiences of being in this long-term remission stage

You will only need to answer the questions that relate to your experience of stopping treatment. The questionnaire will route you to the correct parts, depending on your answers to previous questions.

For example: If at the end of '**Phase I**' you say that you did not go on to stop treatment, you will not have to complete any other parts of the questionnaire.

ABOUT YOU

1. Are you male or female?

1 O Male	2 O Female

2. In what year were you born?

3. In which country do you live?

4. What is the highest degree or level of school you have completed?

1 O No formal qualifications	2 O High school qualifications or diploma	3 O University – Bachelors or Undergraduate degree
4 O University – Masters or PHD	5 O Career or technical qualifications (also known as vocational qualifications)	

5. Where are you usually treated for your CML? Please tick all that apply

1 O Community/family doctor	2 O Hospital	3 O Specialist all cancers centre
4 O CML centre of excellence		5 O Other, please specify below

6. How long have you been living with CML?

1 O Less than 1 year	2 O 1-3 years	3 O 3-5 years
4 O 5-10 years	5 O 10 years or more	

7. Which CML medication have you taken since your diagnosis? Please tick all that apply

1 ○ Imatinib (Glivec/Gleevec or generic Imatinib)
2 ○ Nilotinib (Tasigna)
3 ○ Dasatinib (Sprycel)
4 ○ Bosutinib (Bosulif)
5 ○ Ponatinib (Iclusig)
6 ○ Interferon
7 ○ ABL001/Asciminib
8 ○ Other, please specify below

8. Have you already stopped treatment?

1 ○ I have not yet stopped treatment
2 ○ Yes, between 0-3 months ➔ **Go to question 10**
3 ○ Yes, between 3-6 months ➔ **Go to question 10**
4 ○ Yes, between 6-12 months ➔ **Go to question 10**
5 ○ Yes, between 1-3 years ➔ **Go to question 10**
6 ○ Yes, between 3-5 years ➔ **Go to question 10**
7 ○ Yes, for more than 5 years ➔ **Go to question 10**
8 ○ Yes, but I had to restart ➔ **Go to question 10**

9. What CML medication are you currently taking?

1 ○ Imatinib (Glivec/Gleevec or generic Imatinib)➔ **Go to question 11**
2 ○ Nilotinib (Tasigna) ➔ **Go to question 11**
3 ○ Dasatinib (Sprycel) ➔ **Go to question 11**
4 ○ Bosutinib (Bosulif) ➔ **Go to question 11**
5 ○ Ponatinib (Iclusig) ➔ **Go to question 11**
6 ○ Interferon ➔ **Go to question 11**
7 ○ ABL001/Asciminib ➔ **Go to question 11**
8 ○ Other, please specify below ➔ **Go to question 11**

10. What CML medication were you taking just before you stopped?

1 ○ Imatinib (Glivec/Gleevec or generic Imatinib)	2 ○ Nilotinib (Tasigna)	3 ○ Dasatinib (Sprycel)
4 ○ Bosutinib (Bosulif)	5 ○ Ponatinib (Iclusig)	6 ○ Interferon
7 ○ ABL001/Asciminib		8 ○ Other, please specify below

Phase I (To be completed by all)

These questions are about what considerations, discussion and decisions took place around whether you should stop treatment.

11. How did you **first** hear about the possibility of stopping treatment?

1 O Healthcare professional e.g. physician or nurse
2 O Patient organisation
3 O Family
4 O Printed materials e.g. brochures or leaflets
5 O Media e.g. scientific articles or lay press
6 O Internet
7 O Social media e.g. Facebook or web based group
8 O Other, please specify below

12. What topics did you discuss with your doctor whilst making your decision to try stopping treatment? Please tick all that apply

1 O Benefits of stopping e.g. avoidance of side effects
2 O Risks of stopping e.g. recurrence of CML
3 O Drug withdrawal symptoms
4 O Timing e.g. when it is the best time to stop
5 O Requirements to be met in order to stop treatment e.g. level of remission or duration of treatment
6 O None, I did not discuss with my doctor
7 O Other, please specify below

13. Which topics still concerned you following your discussion with the doctor? Please tick all that apply

- 1 ○ Molecular response criteria when stopping or restarting treatments
- 2 ○ Recurrence of disease (stopping treatment unsuccessful)
- 3 ○ Restarting treatment after unsuccessful stopping
- 4 ○ Frequency of doing PCR tests after stopping
- 5 ○ Choice of medication when restarting treatment
- 6 ○ I did not have any concerns
- 7 ○ Other, please specify below

14. Did you consider switching to a 2nd generation treatment for CML, like Dasatinib or Nilotinib, to achieve a deeper molecular response, allowing you to stop treatment?

- 1 ○ Yes, because stopping treatment is important to me
- 2 ○ Yes, my doctor recommended it
- 3 ○ No, I was satisfied on my current treatment
- 4 ○ No, because I was already on a 2nd generation treatment
- 5 ○ No, I wouldn't want the side effects
- 6 ○ Don't know / not sure
- 7 ○ No, other reason, please specify below

15. 'Would you have considered **adding** an additional treatment to your current treatment, if as a result it allowed you to achieve a deeper molecular response and therefore stop treatment altogether?'

- 1 ○ Yes
- 2 ○ No
- 3 ○ Don't know

16. What are the main reasons that made you consider stopping treatment? Please tick all that apply

1 ○ To get rid of current treatment side effects

2 ○ The fear of side effects caused by long-term treatment

3 ○ Not needing to take medication everyday

4 ○ To see if I can be free of CML without therapy

5 ○ My doctor proposed I join a 'stopping treatment' clinical trial

6 ○ Financial reasons - reduction of costs

7 ○ Planned or unplanned pregnancy

8 ○ Other, please specify below

17. What are the main reasons that made you worry about stopping treatment? Please tick all that apply

1 ○ I didn't have enough information about stopping treatment

2 ○ I wouldn't feel safe going off treatment

3 ○ There is a lack of proper quality PCR monitoring

4 ○ The fear of withdrawal symptoms

5 ○ Recurrence of disease (stopping treatment unsuccessful)

6 ○ I wasn't worried about stopping treatment

7 ○ Other please specify below

18. Did your doctor support your decision to try stopping treatment?

1 ○ Yes

2 ○ No

3 ○ Don't know / can't remember

19. How much did your doctor influence your decision to try stopping treatment?

1 ○ Completely

2 ○ To some extent

3 ○ Not at all

4 ○ Don't know / can't remember

20. Where did you get support and find information about stopping treatment? Please tick all that apply

1 ○ My doctor or other healthcare professional
2 ○ Other CML patients who have stopped treatment
3 ○ Patient organisation
4 ○ Psychological support services
5 ○ Internet
6 ○ Other, please specify below

21. How did this support help you? Please tick all that apply

1 ○ I received useful information
2 ○ I received answers to my questions
3 ○ I received emotional support
4 ○ It did not help me make my decision

22. What information about stopping treatment and therapy-free remission would you have liked to have received?
Please tick all that apply

1 ○ General information on every step of the process of stopping treatment
2 ○ Results from clinical trials investigating stopping treatment
3 ○ Information about required PCR monitoring
4 ○ Expectations in terms of risks and opportunities of stopping treatment
5 ○ Withdrawal symptoms after stopping
6 ○ Side effects on restarting therapy
7 ○ Psychological effects
8 ○ Other (please specify below)

23. Did you proceed to stopping treatment?

1 ○ Yes ➔ **Go to the next section**
2 ○ No ➔ **Thank you this is the end of the questionnaire**

Phase II – Stopping phase: Probation period – To be completed by everyone who went on to try stopping treatment.

Most molecular recurrences (relapses), happen within the first 6 months after stopping treatment, therefore we call this period the ***probation period***.

This section is about your experiences of stopping treatment during the probation period.

**24.** When you stopped treatment did you feel that you were well informed?		
1 ○ Yes, completely	2 ○ Yes, to some extent	3 ○ No, I didn't have all the information I wanted
4 ○ No, but I didn't want to have all the information		

25. How many years were you on medication for CML before stopping?

**26**. How long were you in deep molecular response (at least MR4, or BCR-ABL below 0.01%) before you stopped CML treatment?		
1 ○ I was not in deep molecular response when I stopped	2 ○ Less than 1 year	3 ○ Between 1-2 years
4 ○ Between 2-3 years	5 ○ Between 3-4 years	6 ○ Between 4-8 years
7 ○ More than 8 years	8 ○ Don’t know / can’t remember	

27. Please rate the extent to which you were affected by the following side effects in the weeks **BEFORE** stopping treatment.

PLEASE ONLY RATE THE SIDE EFFECTS YOU EXPERIENCED

1 means the side effect mildly affected your everyday life, and **5** means the side effect completely affected your everyday life.

	1	2	3	4	5
Anxiety	1○	2○	3○	4○	5○
Bruising easily or bleeding	1○	2○	3○	4○	5○
Diarrhoea	1○	2○	3○	4○	5○
Difficulty thinking clearly	1○	2○	3○	4○	5○
Disturbed sleep	1○	2○	3○	4○	5○
Dry mouth	1○	2○	3○	4○	5○
Eye bleeds	1○	2○	3○	4○	5○
Fatigue (tiredness)	1○	2○	3○	4○	5○
Feeling distressed (upset)	1○	2○	3○	4○	5○
Feeling of malaise (not feeling well)	1○	2○	3○	4○	5○
Feeling sad	1○	2○	3○	4○	5○
Hair loss	1○	2○	3○	4○	5○
Headaches	1○	2○	3○	4○	5○
Lack of appetite	1○	2○	3○	4○	5○
Menstrual cycle issues	1○	2○	3○	4○	5○
Muscle soreness or cramping	1○	2○	3○	4○	5○
Nausea	1○	2○	3○	4○	5○
Numbness or tingling	1○	2○	3○	4○	5○
Pain	1○	2○	3○	4○	5○
Rash or skin change	1○	2○	3○	4○	5○
Remembering things	1○	2○	3○	4○	5○
Shortness of breath	1○	2○	3○	4○	5○
Skin pigment changes	1○	2○	3○	4○	5○
Swelling of hands, feet, abdomen, and around eyes	1○	2○	3○	4○	5○
Vomiting	1○	2○	3○	4○	5○
Other (please specify below)	1○	2○	3○	4○	5○

**28.** During the stopping phase did you discuss the following topics with your doctor?	Yes	No
How to deal with withdrawal symptoms	O	O
Response levels and when/if to restart treatment	O	O
How often to monitor the BCR-ABL levels (PCR test)	O	O
Time taken to receive the results of the last PCR test	O	O
How to deal with psychological aspects	O	O
Other please specify below	O	O

29. Did your doctor or another healthcare professional ask you if you were experiencing any physical withdrawal effects during the stopping of treatment?

1 O Yes
2 O No, but I would have liked this
3 O No, but this wasn't necessary
4 O Don't know / can't remember

30. During your stopping phase, how long did you experience withdrawal symptoms for?

1 O I did not experience withdrawal symptoms ➔ **Go to question 33**
2 O For a few days after stopping treatment
3 O For a few weeks after stopping treatment
4 O For a few months after stopping treatment
5 O For a few years
6 O Intermittently – they come and go
7 O Symptoms are still ongoing

31. What withdrawal effects did you experience when treatment was stopped? Please tick all that apply

1 O Pain in muscles, joints or bones
2 O Sweating or skin problems
3 O Depressive episodes or fear or bad mood
4 O Tiredness
5 O Weight loss
6 O Other, please specify below

32. Did your doctor support you in managing all your physical withdrawal effects during the stopping of treatment?

1. ○ Yes, completely
2. ○ Yes, to some extent
3. ○ No, but I would have liked this
4. ○ No
5. ○ Don't know / can't remember

33. During your stopping phase, how often did you feel fear or anxiety?

1. ○ I didn't feel fearful or anxious
2. ○ Daily
3. ○ At least once a week
4. ○ At least once a month
5. ○ Before and/or after PCR monitoring test
6. ○ Less frequently – every few months

34. Did your doctor ask you if you needed psychological support during the stopping of treatment?

1. ○ Yes
2. ○ No, but I would have liked this
3. ○ No but this wasn't necessary
4. ○ Don't know / can't remember

35. Did you receive psychological and/or emotional support during the stopping of treatment?
(This could have been from any source such as a health professional, family or support groups)

1. ○ Yes
2. ○ No, but I would have liked this ➔ **Go to question 37**
3. ○ No but this wasn't necessary ➔ **Go to question 37**
4. ○ Don't know / can't remember ➔ **Go to question 37**

36. What psychological support did you receive? Please tick all that apply

1. ○ Counselling
2. ○ Support from friends and or family
3. ○ Support from patient organisation/s
4. ○ Support from a social media group e.g. Facebook
5. ○ Don't know / can't remember
6. ○ Other

**37.** When treatment was stopped did you experience the following benefits?	Yes	No	Not applicable
Relief from treatment side effects	O	O	O
Relief from the need to remember to take regular medication	O	O	O
Positive impact on finances	O	O	O
Positive impact on emotional well-being	O	O	O
Positive impact on work / education	O	O	O
Positive impact on family and social relationships	O	O	O
Other please specify below	O	O	O

38. After stopping treatment, **on average**, how often were you monitored by your doctor by a PCR test in the first 6 months?

1 O Every week
2 O Every month
3 O Every 2 months
4 O Every 3 months
5 O Every 6 months

39. After stopping treatment, how long did it take (on average) between your blood tests and until you heard about the result of the PCR?

1 O Less than 7 days
2 O Between 7-14 days
3 O Between 15-30 days
4 O Longer than 30 days
5 O Don't know / Can't remember

40. After stopping treatment, were you satisfied with how often your CML was monitored?

1 O Yes, frequency of testing was about right
2 O No, testing was too frequent
3 O No, testing was too infrequent
4 O Don't know / Can't remember

41. Following stopping treatment, did the disease reoccur and treatment have to restart?

1 O Yes ➔ **Go to Section 'Phase IIIA – Restarting treatment'**
2 O No, but I am still in the probation period➔ **Go to question 69**
3 O No, I am in long term remission➔ **Go to Section 'Phase IIIB – Long term remission'**

Phase IIIA – Restarting treatment

We would like to know more about the restarting treatment stage that happened following the stopping of a TKI treatment, and subsequent reoccurrence of the disease.

42. How long after stopping a TKI did the disease reoccur, and treatment had to restart?

- 1 ○ Within the first month
- 2 ○ After 2-3 months
- 3 ○ After 3-6 months
- 4 ○ After 6-12 months
- 5 ○ After more than 1 year
- 6 ○ After more than 2 years
- 7 ○ After more than 3 years

43. When being told your disease had reoccurred, and you had to restart treatment, was this explained in a way you could understand?

- 1 ○ Yes completely
- 2 ○ Yes, to some extent
- 3 ○ No, I did not understand the explanation
- 4 ○ I did not have an explanation but would have liked one
- 5 ○ I did not need / want an explanation
- 6 ○ Don't know / can't remember

**44.** To what extent do you agree or disagree with the following statements?<br>When I was first told my disease had reoccurred I felt….	Strongly agree	Agree	Neither agree nor disagree	Disagree	Strongly Disagree
scared / anxious	○	○	○	○	○
disappointed	○	○	○	○	○
depressed	○	○	○	○	○
confused	○	○	○	○	○
relieved	○	○	○	○	○

45. Before your treatment restarted were your treatment options explained to you?

- 1 ○ Yes completely
- 2 ○ Yes, to some extent
- 3 ○ No
- 4 ○ Don't know / can't remember

46. What information was provided to you when you were told that you had to restart the treatment? Please tick all that apply

- 1 ○ That the same treatment taken before stopping would restart
- 2 ○ That another treatment would be started (switching to other TKI)
- 3 ○ Information on possible side effects
- 4 ○ Treatment goals and expectations when to regain remission
- 5 ○ The timing of when treatment would restart
- 6 ○ The experiences of other patients who had restarted treatment
- 7 ○ Potential for another attempt to stop treatment in the future
- 8 ○ Other, please specify below

47. Who provided you with the necessary information? Please tick all that apply

- 1 ○ Treating doctor
- 2 ○ Nurse or other healthcare professional
- 3 ○ Patient organisation
- 4 ○ Other patients
- 5 ○ Family members
- 6 ○ Other, please specify below
- 7 ○ Internet

48. Did you restart on the same treatment that you had been taking before stopping?

- 1 ○ Yes, I took the same treatment again
  ➔ **Go to question 50**
- 2 ○ Yes, I took the same treatment again, but with a different dose
  ➔ **Go to question 50**
- 3 ○ No, I switched to a different treatment

49. Which treatment did you switch to? If you were put on a combination of drugs, please tick both names.

- 1 ○ Imatinib (Glivec/Gleevec or generic Imatinib)
- 2 ○ Nilotinib (Tasigna)
- 3 ○ Dasatinib (Sprycel)
- 4 ○ Bosutinib (Bosulif)
- 5 ○ Ponatinib (Iclusig)
- 6 ○ Interferon
- 7 ○ ABL001/Asciminib
- 8 ○ I wasn't restarted on any treatment
- 9 ○ Don't know / not sure
- 10 ○ I was restarted on another treatment not listed here, please specify

**50.** In the first weeks after restarting the treatment, how did you feel **physically**?		
1 O Worse than before stopping treatment	2 O Worse than during stopping treatment	3 O The same as before stopping treatment
4 O Better than during stopping treatment	5 O Better than before stopping treatment	

**51.** In the first weeks after restarting the treatment, how did you feel **emotionally**?		
1 O Worse than before stopping treatment	2 O Worse than during stopping treatment	3 O The same as before stopping treatment
4 O Better than during stopping treatment	5 O Better than before stopping treatment	

52. Please rate the extent to which you were affected by the following side effects when you **RESTARTED** treatment.

PLEASE ONLY RATE THE SIDE EFFECTS YOU EXPERIENCED

***1** means the side effect mildly affected your everyday life, and **5** means the side effect completely affected your everyday life.*

	1	2	3	4	5
Anxiety	1○	2○	3○	4○	5○
Bruising easily or bleeding	1○	2○	3○	4○	5○
Diarrhoea	1○	2○	3○	4○	5○
Difficulty thinking clearly	1○	2○	3○	4○	5○
Disturbed sleep	1○	2○	3○	4○	5○
Dry mouth	1○	2○	3○	4○	5○
Eye bleeds	1○	2○	3○	4○	5○
Fatigue (tiredness)	1○	2○	3○	4○	5○
Feeling distressed (upset)	1○	2○	3○	4○	5○
Feeling of malaise (not feeling well)	1○	2○	3○	4○	5○
Feeling sad	1○	2○	3○	4○	5○
Hair loss	1○	2○	3○	4○	5○
Headaches	1○	2○	3○	4○	5○
Lack of appetite	1○	2○	3○	4○	5○
Menstrual cycle issues	1○	2○	3○	4○	5○
Muscle soreness or cramping	1○	2○	3○	4○	5○
Nausea	1○	2○	3○	4○	5○
Numbness or tingling	1○	2○	3○	4○	5○
Pain	1○	2○	3○	4○	5○
Rash or skin change	1○	2○	3○	4○	5○
Remembering things	1○	2○	3○	4○	5○
Shortness of breath	1○	2○	3○	4○	5○
Skin pigment changes	1○	2○	3○	4○	5○
Swelling of hands, feet, abdomen, and around eyes	1○	2○	3○	4○	5○
Vomiting	1○	2○	3○	4○	5○
Other	1○	2○	3○	4○	5○

53. Overall, when you restarted, how did side effects compare to before you stopped treatment?

1 O The side effects after restarting treatment were worse
2 O The side effects after restarting treatment were not as bad
3 O The side effects were about the same
4 O I experienced different side effects than those before stopping

**54.** To what extent do you agree or disagree with the following statements?	Strongly agree	Agree	Neither agree nor disagree	Disagree	Strongly Disagree
I feel my disease is under control again now I have restarted treatment	O	O	O	O	O
I feel my disease is better monitored when I have treatment	O	O	O	O	O

55. Did you receive psychological and/or emotional support when you restarted your treatment? (This could have been from any source such as a health professional, family or support groups)

1 O Yes
2 O No, but I would have liked this ➔ **Go to question 57**
3 O No but this wasn't necessary ➔ **Go to question 57**
4 O Don't know / can't remember ➔ **Go to question 57**

56. What psychological support did you receive? Please tick all that apply

1 O Counselling
2 O Support from friends and or family
3 O Support from patient organisation/s
4 O Support from a social media group e.g. Facebook
5 O Don't know / can't remember
6 O Other

57. Do you think you will consider stopping your treatment again in the future?

1 ○ Yes, I have already stopped treatment again and it has been successful ➔ **Go to Section 'Phase IIIB – Long term remission'**

2 ○ Yes, if my doctor suggests it ➔ **Go to question 69**

3 ○ Yes, but I will wait for a longer time frame whilst I am on treatment ➔ **Go to question 69**

4 ○ Yes, but I want to wait until experts know much more about the safety and success factors of stopping treatment ➔ **Go to question 69**

5 ○ No, I don't want to try to stop again in the mid-term ➔ **Go to question 69**

6 ○ Don't know/not sure

7 ○ Other, please specify below ➔ **Go to question 69**

Phase IIIB – Stopping phase: Long-term therapy-free remission

We would like to know a little bit about the long-term remission stage that follows stopping of a TKI treatment.

**58.** What are your major concerns about being in Treatment Free Remission? Please tick all that apply		
1 ○ I don't have any concerns	2 ○ Late recurrence of the CML	3 ○ Late detection of recurrence
4 ○ Uncertainty about the future in terms of CML	5 ○ More frequent PCR tests than before stopping	7 ○ Other (please specify below)
6 ○ Misunderstanding of people thinking that I am now cured		

**59.** What would you change about your experience of stopping treatment? Please tick all that apply		
1 ○ To be treated by a doctor who has a more experience in stopping treatment	2 ○ Quicker communication of PCR test results to patients	3 ○ To be in contact with other patients in the same phase of stopping treatment (decision phase, stopping phases)
4 ○ Better understanding of the current experts' knowledge of stopping treatment		5 ○ Other (please specify below)

**60.** How often are you monitored by your doctor by a PCR test?		
1 ○ Every week	2 ○ Every month	3 ○ Every 2 months
4 ○ Every 3 months	5 ○ Every 6 months	6 ○ Once a year

**61.** How do you feel about the frequency of monitoring / PCR tests whilst in Treatment Free Remission (TFR)?		
1 ○ Testing is too frequent	2 ○ Frequency of tests is about right	3 ○ Testing is too infrequent

62. Do you keep to the monitoring schedule set by your doctor?

- 1 O Always
- 2 O Often / Most of the time
- 3 O Occasionally / Sometimes
- 4 O Rarely
- 5 O Never

63. How concerned are you with fluctuations (PCR going up and down) / changes in your PCR test results?

- 1 O Not at all concerned
- 2 O Slightly concerned
- 3 O Somewhat concerned
- 4 O Moderately concerned
- 5 O Extremely concerned

64. Do you receive psychological and/or emotional support?
(This can be from any source such as a health professional, family or support groups)

- 1 O Yes
- 2 O No, but I would like this ➔ **Go to question 66**
- 3 O No but this isn't necessary ➔ **Go to question 66**

65. What psychological support do you receive? Please tick all that apply

- 1 O Counselling
- 2 O Support from friends and or family
- 3 O Support from patient organisation/s
- 4 O Support from a social media group e.g. Facebook
- 5 O Other

66. Overall, do you feel you receive adequate care in this Treatment Free Remission phase?

- 1 O Yes, completely
- 2 O Yes, to some extent
- 3 O No

67. How could your doctor improve your experience of stopping treatment? Please tick all that apply

- 1 O No improvement necessary
- 2 O More information on current data, in an easy to understand language
- 3 O Better doctor / patient relationship
- 4 O Better PCR monitoring
- 5 O Better psychological support
- 6 O Other (please specify below)

68. Do you feel you are still a CML patient?

- 1 O Yes, I will always be a CML patient because I do not feel I am completely cured
- 2 O Yes, I feel like a CML patient but my disease doesn't play much of a role in my everyday life any more
- 3 O No, I know I am not cured but I don't feel like a CML patient anymore
- 4 O No, I feel I am cured from CML
- 5 O Other (please specify below)

FINAL SECTION

69. What advice would you give to a patient that is considering stopping treatment? Please tick all that apply

- 1 O Look for the best doctor with experience in stopping treatment
- 2 O Always be well informed about your PCR results and treatment options
- 3 O Look for simple and good information about each step of stopping treatment
- 4 O Talk with other patients who have stopped, or are considering stopping
- 5 O Receive information from patient organisations about stopping treatment
- 6 O Other (please specify below)
- 7 O Get psychological support
- 8 O Get emotional support
